# Supplementary material for: Spatial Variation in Stream-Water Bacterial Communities Across Sites Differing in Wild Chinese Giant Salamander Detection Frequency
Source: Biology (Basel). 2026 Sep 18;15(18):1647. doi: 10.3390/biology15181647 (PMC13604818; doi:10.3390/biology15181647)
Supplement: Supplementary file 1 [file biology-15-01647-s001.zip › Data Related to “Spatial Variation in Stream-Water Bacterial Communities across Sites Differing in Wild Chinese Giant Salamander Detection Frequency”/Microbial Data/result/03.TaxonVisual/ternary/sample/genus/S1-S3-S6.ternary.pdf]

ternaryplot

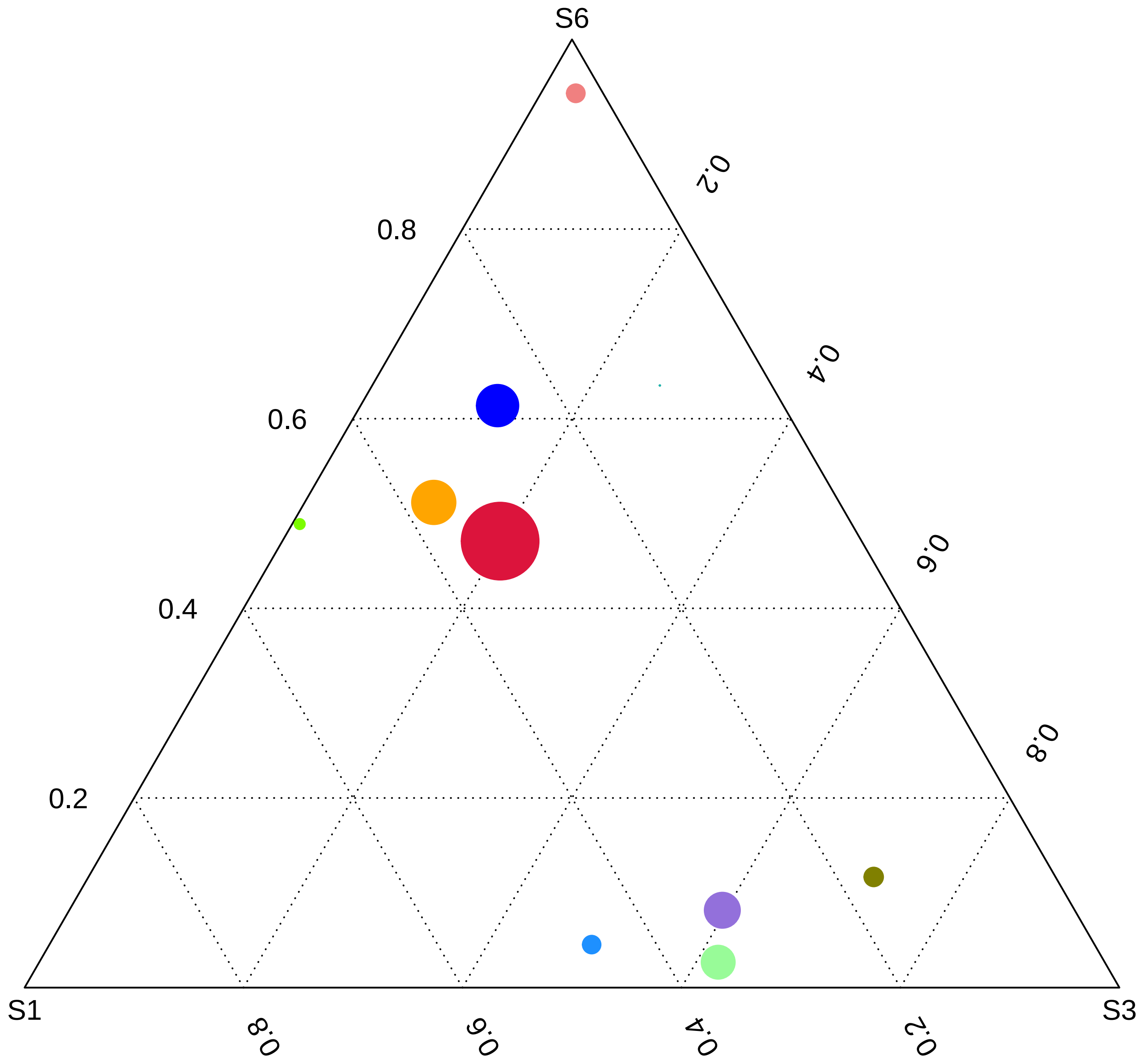

- unclassified_Chloroplast
- Flavobacterium
- Tychonema_CCAP_1459−11B
- Rhodoferax
- Arenimonas
- Brevundimonas
- Polaromonas
- Luteolibacter
- unclassified_Microscillaceae
- Cypionkella
